# Alopurinol versus Trimetazidina como Antianginal: Um Ensaio Clínico Randomizado

**DOI:** 10.36660/abc.20240500

**Published:** 2024-09-06

**Authors:** Luiz Antonio Machado Cesar, Bruno Mahler Mioto

**Affiliations:** 1 Instituto do Coração do Hospital das Clínicas Faculdade de Medicina Universidade de São Paulo São Paulo SP Brasil Instituto do Coração do Hospital das Clínicas da Faculdade de Medicina da Universidade de São Paulo, São Paulo, SP – Brasil

**Keywords:** Trimetazidina, Ensaio Clínico Controlado, Alopurinol

O primeiro caso de tratamento de angina de peito com uso de medicamento foi descrito pelo professor T. Lauder Brunton^1^ em 1857, em Edimburgo. O professor Brunton documentou as experiências dos pacientes com “intensa ansiedade ao ter o peito comprimido” e prontamente aliviado pelo nitrito de amila. Quase um século depois, Mason et al.^2^ demonstraram elegantemente os efeitos desse vasodilatador volátil em homens em 1965. Além de intensos efeitos vasodilatadores nos sistemas arteriolar e venoso, uma intensa resposta adrenérgica foi desencadeada pela hipotensão que se seguiu. O uso de nitratos orgânicos começou em 1946,^3^ seguido de betabloqueadores (BBs) na década de 1960,^4^ estabelecendo-os como base para prevenção, ou para reduzir episódios de angina e aliviar a dor usando fórmulas sublinguais ou orais de nitratos. Uma combinação de BBs com nitratos orais de ação prolongada foi usada para evitar angina por muitos anos. Devido ao seu efeito protetor contra isquemia e arritmia ventricular, os BBs foram reforçados como os medicamentos antianginosos de primeira escolha após serem testados em pacientes com infarto agudo do miocárdio (IAM) durante as décadas de 1980 e 1990.

Os resultados dos ensaios^5,6^mostraram uma redução relativa de 15–20% na incidência de IAM e na mortalidade cardiovascular durante o período de acompanhamento de até 2,5 anos. Mais recentemente, o ensaio COMMIT^7^ com tartarato de metoprolol reduziu a mortalidade apenas em 1% (risco relativo). Além disso, existem preocupações quanto à duração dos efeitos, especialmente em pacientes com fração de ejeção do ventrículo esquerdo (FEVE) normal. Em pacientes com síndrome coronariana crônica (SCC) e FEVE preservada, nenhum estudo testou os BBs na redução de eventos cardiovasculares. Recentemente, registros de *big data* permitiram abordagens estatísticas utilizando escores de propensão para testar resultados que não apoiam o uso de BBs como medida preventiva contra mortalidade ou IAM em pacientes mesmo naqueles 1 ano após IAM.^8,9^ Um ensaio publicado recentemente^10^ testou metoprolol ou bisoprolol após IAM, em pacientes com FEVE normal; com período médio de acompanhamento de 3,5 anos; não foram observadas vantagens em relação à mortalidade ou à incidência de infarto do miocárdio. Portanto, precisamos repensar o uso generalizado de BBs na SCC. Os medicamentos antianginosos mais recentes incluem nicorandil, trimetazidina, ivabradina e ranolazina, que foram testados em pacientes com angina que já estavam recebendo terapia com BB.

Antes, antagonistas do cálcio foram testados na década de 1970^11^ e provaram ser eficazes no controle de espasmos coronarianos e na promoção da microvasodilatação coronariana. Assim, foram administrados com BBs para tratar a angina como segundo medicamento (dihidropiridinas). Notavelmente, é vantajoso combinar um BB com aqueles sem quaisquer efeitos hemodinâmicos, como trimetazidina, ranolazina e alopurinol. A trimetazidina bloqueia o 3-cetoacil coenzima A tiolase de cadeia longa e muda o metabolismo energético cardíaco da oxidação de ácidos graxos livres para a oxidação da glicose via piruvato que gera 20% mais trifosfato de adenosina através do ciclo de Krebs para cada molécula de oxigênio no processo.^12^ Estudos randomizados compararam trimetazidina com placebo para SCC desde 2000, e todos eles envolveram pacientes que já tomavam BBs e/ou outros medicamentos antianginosos. Os resultados mostraram uma redução robusta e significativa da angina e melhoria da qualidade de vida.^13,14^

Além disso, existem estudos observacionais em milhares de pacientes com angina classe 2 ou 3 que já recebem outras terapias com redução significativa da angina quando a trimetazidina foi combinada com BBs.^15^ Em contrapartida, existem poucos estudos com alopurinol. Existem dois pequenos ensaios clínicos com alopurinol.^16,17^ Ambos os resultados foram controversos. O alopurinol permitiu um maior tempo de angina no teste ergométrico, enquanto a ranolazina levou a um maior tempo de exercício até a depressão do segmento ST, caso contrário, o oposto foi observado no estudo em comparação com o placebo.

Nesta edição dos Arquivos Brasileiros de Cardiologia, o ensaio clínico de Viana et al.^18^ compara alopurinol e trimetazidina em um estudo randomizado e cego, o que nunca foi feito antes para pacientes com SCC e angina. O principal resultado foi melhoria da qualidade de vida e redução da angina. Os resultados mostram uma melhoria na qualidade de vida com ambos os medicamentos desde o início até a avaliação final; entretanto, a trimetazidina foi superior ao alopurinol, principalmente na redução da angina, conforme visto na Figura 2 e Tabela 2 do manuscrito. A angina foi reduzida em 33–66% com trimetazidina em comparação com 25–40% com alopurinol. Conforme observado pelos autores, a principal limitação do estudo é o placebo defeituoso para cegar os pacientes e evitar o efeito placebo de qualquer um dos dois medicamentos. Além disso, este estudo mostra a variabilidade do efeito do alopurinol no tratamento da angina, e acredito que eles perderam a oportunidade de comparar o alopurinol apenas com o BB e não com dois antianginosos já em uso. No entanto, este estudo incluiu mais pacientes do que os anteriores com alopurinol para angina. Porém, perdeu a oportunidade de realizar o teste ergométrico, que é o método padrão ouro para avaliar o tempo até a angina, o tempo até o ST e a angina limitante. Esses estudos com o alopurinol o tornam um medicamento raramente prescrito para tratar a angina, que pode ser tratado de forma mais eficaz com outros medicamentos. Além disso, não temos um estudo comparando medicamentos antianginosos metabólicos como primeira combinação com um BB contra medicamentos hemodinâmicos, como bloqueadores dos canais de cálcio e nitratos de ação prolongada. Assim, continuamos a tratar pacientes com angina e doença coronariana obstrutiva primeiro com BB e introduzindo um segundo medicamento para melhorar os sintomas quando necessário. Além disso, consideramos a frequência cardíaca, a pressão arterial e a presença de disfunção ventricular esquerda para determinar o segundo medicamento mais adequado para alcançar melhor tolerabilidade e qualidade de vida em nossos pacientes. Concluindo, não podemos desconsiderar a combinação de BBs com medicamentos que tenham efeitos intracelulares para aliviar os episódios estressantes da angina de peito ou quando a angina persiste ou reaparece apesar da realização da revascularização.
