# Supplementary material for: Alopurinol versus Trimetazidina como Antianginal: Um Ensaio Clínico Randomizado
Source: Arq Bras Cardiol. 2024 Sep 6;121(8):e20240500. [Article in Portuguese] doi: 10.36660/abc.20240500 (PMC11495810; doi:10.36660/abc.20240500)
Supplement: Translation

# Translation: Allopurinol versus Trimetazidine as Antianginal: A Randomized Clinical Trial

**DOI:** 10.36660/abc.20240500i

**Authors:** Luiz Antonio Machado Cesar, Bruno Mahler Mioto

**Affiliations:** 1 Brazil Instituto do Coração do Hospital das Clínicas da Faculdade de Medicina da Universidade de São Paulo, São Paulo, SP – Brazil

**Keywords:** Trimetazidine, Controlled Clinical Trial, Allopurinol

The first instance of angina pectoris treatment using a drug was described by Professor T Lauder Brunton^1^ in 1857 in Edinburgh. Professor Brunton documented patients’ experiences with “*intense anxiety of having their chest compressed*” and alleviated promptly by amyl nitrite. Almost a century later, Mason et al.^2^elegantly demonstrated the effects of this volatile vasodilator in men in 1965. In addition to its intense vasodilator effects on the arteriolar and venous systems, an intense adrenergic response was elicited by hypotension that followed. The use of organic nitrates started in 1946,^3^ followed by beta-blockers (BBs) in the 1960s,^4^ establishing them as a basis for preventing or reducing angina episodes and relieving pain using sub-lingual or oral spray formulas of nitrates. A combination of BBs with oral long-acting nitrates was used to avoid angina for many years. Due to its protective effect against ischemia and ventricular arrhythmia, BBs were reinforced as the first-choice antianginal drugs after being tested in patients with acute myocardial infarction (AMI) during the 1980s and 1990s.

The results of the trials^5,6^showed a relative reduction of 15–20% in the AMI incidence and cardiovascular mortality during the follow-up period up to 2.5 years. More recently, the COMMIT trial^7^ with metoprolol tartrate only reduced mortality by 1%(relative risk). Besides, there are concerns regarding how long the effects shall last, especially in patients with normal left ventricular ejection fraction (LVEF). In patients with chronic coronary syndrome (CCS) and preserved LVEF, no trial tested BBs in the reduction of cardiovascular events. Recently big data registries allowed statistical approaches using propensity scores to test with results that do not support the use of BBs as a preventive measure against mortality or AMI in patients even in those 1 year after AMI.^8,9^ A recently published trial^10^tested metoprolol or bisoprolol after AMI, in patients with normal LVEF; with a median follow-up period of 3.5 years; no advantages were observed regarding mortality or myocardial infarction incidence. Therefore, we need to re-think the widespread use of BBs in CCS. The more recent antianginal drugs include nicorandil, trimetazidine, ivabradine, and ranolazine, which were tested in patients with angina who were already receiving BB therapy.

Before, calcium antagonists were tested in the 1970s^11^ and proven effective in controlling coronary spasms and promoting coronary micro-vasodilation. Hence, they were administered with BBs to treat angina as the second drug (dihydropyridines). Notably, it is advantageous to combine a BB with those without any hemodynamic effects, such as trimetazidine, ranolazine, and allopurinol. Trimetazidine blocks the long-chain 3-ketoacyl coenzyme A thiolase and shifts cardiac energy metabolism from free fatty acid oxidation to glucose oxidation via pyruvate that generates 20% more adenosine triphosphate through the Krebs cycle for each oxygen molecule in the process.^12^ Randomized studies have compared trimetazidine with placebo for CCS since 2000, and all of them involved patients already on BBs and/or other antianginal drugs. The results showed a robust and significant reduction in angina and improved quality of life.^13,14^ Furthermore, there are observational studies on thousands of patients with class 2 or 3 angina already receiving other therapies with a significant reduction in angina when trimetazidine was combined with BBs.^15^ In contrast, there are only a few studies with allopurinol. There are two small clinical trials with allopurinol.^16,17^ Both results were controversial. Allopurinol allowed a longer time to angina in the treadmill test, whereas ranolazine led to a longer exercise time until ST depression, otherwise the opposite was observed in the study compared with placebo.

In this issue of *Arquivos Brasileiros de Cardiologia*, the clinical trial by Viana et al.^18^ compares allopurinol and trimetazidine in a randomized blinded study, which has never been done before for patients with CCS and angina. The main outcome was improved quality of life and reduction of angina. The results show an improved quality of life with both drugs from baseline to final evaluation; however, trimetazidine was superior to allopurinol, especially in reducing angina as seen in Figure 2 and Table 2 of the manuscript. Angina reduced by 33–66% with trimetazidine compared to 25–40% with allopurinol. As noted by the authors, the major limitation of the study is the faulty placebo to make patients blinded and avoid a placebo effect of any of the two drugs. Besides, this study shows the variability of the effect of allopurinol to treat angina, and I believe that they missed an opportunity to compare allopurinol only on top of BB rather than with two antianginal drugs already being used. Nevertheless, this study did include more patients than previous ones with allopurinol for angina. However, it missed the opportunity to conduct the treadmill test, which is the gold standard method to evaluate time to angina, time to ST, and limiting angina. These studies with allopurinol make it a drug rarely prescribed to treat angina, which can be treated more effectively with other drugs. Furthermore, we do not have a study comparing metabolic antianginal drugs as the first combination with a BB against hemodynamic drugs such as calcium channel blockers and long-acting nitrates. Hence, we continue to treat patients with angina and obstructive coronary disease first with a BB and introduce a second drug to ameliorate symptoms when necessary. Additionally, we consider heart rate, blood pressure, and the presence of left ventricular dysfunction to determine the most appropriate second drug to achieve better tolerability and quality of life in our patients. In conclusion, we cannot disregard the combination of BBs with drugs that have intracellular effects to alleviate the stressful episodes of angina pectoris or when angina persists or reappears despite performing revascularization.
